# Dadaist2: A Toolkit to Automate and Simplify Statistical Analysis and Plotting of Metabarcoding Experiments

**DOI:** 10.3390/ijms22105309

**Published:** 2021-05-18

**Authors:** Rebecca Ansorge, Giovanni Birolo, Stephen A. James, Andrea Telatin

**Affiliations:** 1 Gut Microbes and Health Programme, Quadram Institute Bioscience, Norwich NR4 7UQ, UK; rebecca.ansorge@quadram.ac.uk (R.A.); steve.james@quadram.ac.uk (S.A.J.); 2 Medical Sciences Department, University of Turin, 10126 Turin, Italy; giovanni.birolo@unito.it

**Keywords:** bioinformatics, metabarcoding, microbial communities, amplicon sequence variant, exact amplicon variant, bacterial taxonomy, visualizations, statistical analysis, numerical ecology

## Abstract

The taxonomic composition of microbial communities can be assessed using universal marker amplicon sequencing. The most common taxonomic markers are the 16S rDNA for bacterial communities and the internal transcribed spacer (ITS) region for fungal communities, but various other markers are used for barcoding eukaryotes. A crucial step in the bioinformatic analysis of amplicon sequences is the identification of representative sequences. This can be achieved using a clustering approach or by denoising raw sequencing reads. DADA2 is a widely adopted algorithm, released as an R library, that denoises marker-specific amplicons from next-generation sequencing and produces a set of representative sequences referred to as ‘Amplicon Sequence Variants’ (ASV). Here, we present Dadaist2, a modular pipeline, providing a complete suite for the analysis that ranges from raw sequencing reads to the statistics of numerical ecology. Dadaist2 implements a new approach that is specifically optimised for amplicons with variable lengths, such as the fungal ITS. The pipeline focuses on streamlining the data flow from the command line to R, with multiple options for statistical analysis and plotting, both interactive and automatic.

## 1. Introduction

High-throughput amplicon sequencing of taxonomic markers is a cost-effective and widely adopted method for determining the composition of mixed natural communities. In addition to the analysis of microbial communities using standard marker sequences such as 16S rDNA or the fungal internal transcribed spacer (ITS) region, there are also applications targeting eukaryotic genes (e.g., *trnL* for chloroplasts, COI for mitochondria, or nuclear 18S rDNA) that decipher the composition of environmental, host-associated, or manufactured food-associated microbiomes [1]. Amplicon sequencing detects the presence of molecular species (barcode sequences) and uses that information to infer the presence of microorganisms under a set of assumptions and caveats. These include technical biases arising from the amplification procedure that are prone to introducing errors and chimeras, making accurate community description and between-study comparisons challenging. The natural occurrence of multiple copies of a marker sequence, such as 16S, in an organism’s genome, further complicates taxonomic affiliation and abundance inference. Addressing these caveats, in both laboratory and bioinformatic data processing, often poses a challenge for reproducible and comparable amplicon-based microbiome studies [2].

The vast majority of studies focus on the bacterial 16S sequence and this is reflected in the abundance of protocols and optimizations for improving and standardizing 16S amplicon sequencing. One advantage of the 16S sequence is that the target regions are highly conserved in length. Therefore, amplicon sizes are designed to allow merging of the two paired reads to obtain one long sequence covering the entire region. The length of other markers, such as the ITS sequence, is highly variable, posing additional challenges [3]. While there are some attempts to accommodate so-called ‘primer read-through’ in shorter amplicons, there are currently few options for the analysis of amplicons that are too long for the merging of paired reads. Mechanisms to address both these issues are lacking in automatic pipelines.

Molecular species can be defined from amplicon sequences by clustering ‘operational taxonomic units’ (OTUs) or by reconstructing (‘denoising’) ‘amplicon sequence variants’ (ASVs) [4,5,6]. A major challenge for amplicon experiments is to go from raw data to biologically informative data in a representable format. For both aspects, there are well-tested and widely-adopted tools such as DADA2 (Divisive Amplicon Denoising Algorithm, version 2) [6], Qiime2 (Quantitative Insights Into Microbiome Ecology) [7,8], DadaSnake [9] and LotuS [10] for analysis of amplicon data; and PhyloSeq [11], and Rhea [12] and MicrobiomeAnalyst [13] for downstream visualization and statistical analysis of the results. Using multiple tools, guidelines and coding languages for each step of the analyses complicates reproducibility and automatization, and leads to the need for error-prone format conversions. In particular, bridging the step from quality control and sequence analyses (primary analysis), to publication-ready comparative representation and statistics (secondary analysis) can be challenging. In most experiments, this includes importing amplicon results and metadata into R and combining this information to allow appropriate numerical ecology and statistics, such as taxonomic profiles and diversity indices. Currently, scientists analysing amplicon experiments need to perform this step manually and by themselves to access downstream analysis routes for retrieving biologically informative results.

**Figure 1:**
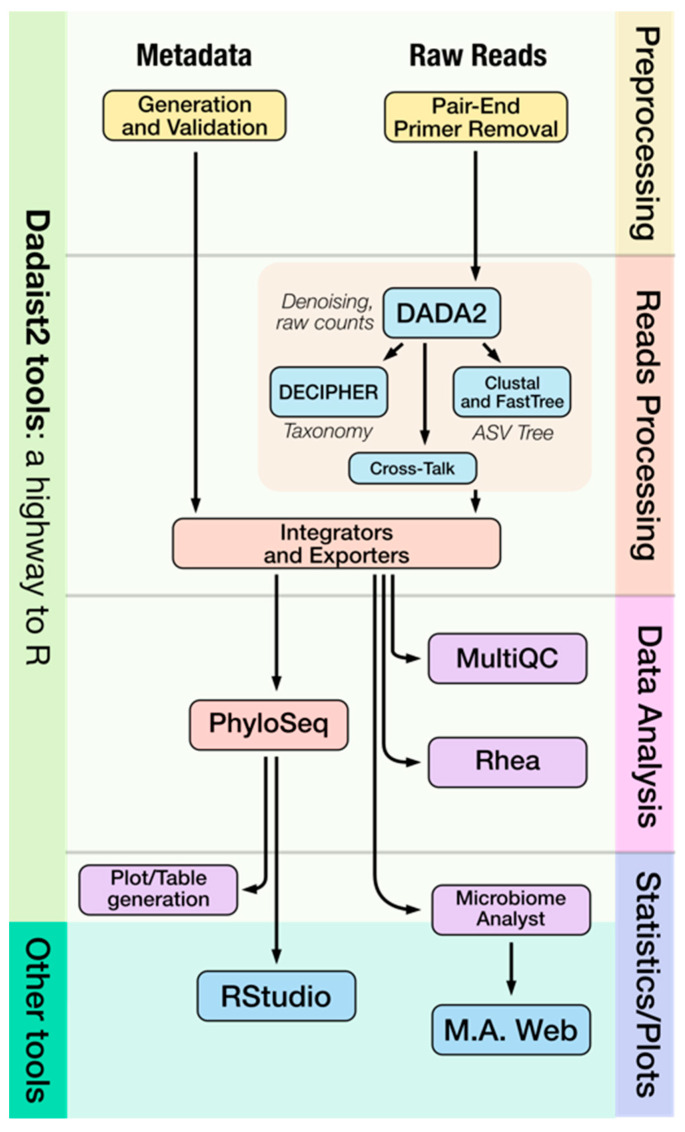
Dadaist2 provides both a workflow for primary analysis, based on DADA2, and a set of integrators and exporters to enable a range of secondary analyses, enabling both user-friendly exploration and reproducible statistical analyses.

Here, we present Dadaist2, a command-line-based application that easily implements the use of DADA2 with a dedicated workflow for ITS profiling (Figure 1) and that focuses on filling the gap between primary and secondary analysis, streamlining the numerical ecology analysis that can be performed manually (as a result of the automatic creation of a PhyloSeq object), or interactively using MicrobiomeAnalyst (thanks to the automatic generation of compatible input files).

The bioinformatic analysis of microbiome studies is a good example of complex workflows that rely on a range of tools and require several data types to be coherently manipulated. This complexity can be managed using different approaches. For example, the developers of Qiime2 re-engineered the whole workflow and introduced an original archive format to ensure that the correct filetypes are provided. This solution is effective but still leaves a gap at the end of the analysis. In contrast, USEARCH (another popular tool that performs the denoising of amplicons via the Unoise3 algorithm) provides several functions in a more traditional command line interface, which is more error-prone, and still requires a lot of steps to be executed, and great to be taken at every step to ensure consistency between the files produced [14,15].

DadaSnake is a workflow written in SnakeMake [16] which wraps DADA2 denoising, but involves a more complex installation and mainly focuses on the primary analysis (with limited support for downstream numerical ecology).

Dadaist2 is designed to provide a familiar experience of commonly used command line tools (like USEARCH), while including more automatic controls to prevent accidental errors. Dadaist2 simplifies the execution of R packages from the command line (like DadaSnake), is easy to install with Miniconda (like Qiime2), has both a single command for full analysis and a set of independent modules that can be executed to perform a single action, and helps the users exploring their dataset with a range of options. Secondary analysis is enabled using different solutions that can be adapted to different user expertise levels and include a web-based exploration of the experiment’s diversity. A feature comparison of Dadaist with two alternative workflows using DADA2 (the very popular Qiime2 framework and the specialized DadaSnake pipeline) is provided in Table 1.

The execution of a multistep workflow can be difficult to inspect when errors are encountered, both from a technical point of view (e.g., insufficient memory) and from an experimental point of view (e.g., low-quality reads). To assist the user in the troubleshooting of both these aspects, Dadaist2 produces a user-friendly execution log in HTML format and a MultiQC report summarizing the most relevant aspects of the experiment.

## 2. Results

**Figure 2:**
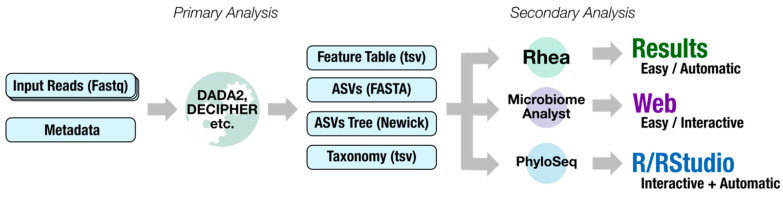
Simplified data flow from raw sequencing reads to the final results, split into two main parts. Primary analysis (traditionally performed from the command line) generates a raw count matrix (feature table), a set of representative sequences of molecular diversity (ASVs), and a phylogenetic tree of the sequences and the predicted taxonomy for each ASV. In the secondary analysis, these intermediates are visualised and used to test hypotheses and generate summaries. Dadaist2 not only performs the primary analysis, but also streams the data into different scenarios for secondary analyses: a fully automated protocol (Rhea), a user-friendly and interactive web platform for the analysis of microbiome data (MicrobiomeAnalyst), and the generation of a convenient PhyloSeq object for further downstream analyses (that can be both interactive, via R, or non-interactive as Rscripts).

Dadaist2 is both a set of modular tools (to build or extend existing pipelines) and a complete pipeline for amplicon sequencing analysis beginning with the raw reads (Figure 1). The pipeline can be divided into primary analysis, including newly implemented modules for raw data analysis, and secondary analysis streamlining the ‘hand-off’ of results to numerical ecology statistics and data visualization (Figure 2). This produces data in suitable formats suitable for direct used with tools that allow the creation of publication-ready results via PhyloSeq (for users with knowledge of R), or MicrobiomeAnalyst (for users without any prior experiences with R), and a fully automated analysis implementing the Rhea protocol. Dadaist2 provides an easy and user-friendly interface between data-mingling and data-representation, without the need for multiple coding languages.

### 2.1. Dadaist2 Primary Analysis

Raw reads are pre-processed using Fastp or Cutadapt [17,18], and subsequently denoised using DADA2. Taxonomy annotation can be achieved using either DADA2 or DECIPHER [19]. For this, common reference databases can be downloaded directly within Dadaist2. The main output of the primary analysis is a coherent set of files that include representative sequences, a feature table, a phylogenetic tree and the associated metadata. To be further analysed in the secondary analysis, all these files are automatically assembled into a PhyloSeq object, to simplify the time-consuming and error-prone task of importing all the required data in the correct format.

#### 2.1.1. Cross Talk Removal

High-throughput amplicon experiments usually result in highly multiplexed sequencing runs where each sample is identified by a molecular barcode that is also sequenced. De-multiplexing errors can cause reads to be assigned to the wrong sample [20]. While the effect is often negligible, it may affect analysis if a fraction of reads leak from a high-abundance sample to a low- or even zero-abundance sample; this would affect diversity rate estimation. The removal of crosstalk between samples in feature tables has been addressed previously by the UNCROSS2 algorithm [21] implemented in closed-source USEARCH software [15]. In Dadaist2, we include the first open-source implementation of the UNCROSS2 algorithm.

#### 2.1.2. Dedicated Workflow for Amplicons of Variable Lengths

In experiments where both paired-end reads are consistently overlapping, a standard DADA2 workflow is adopted, but for sequencing runs, where it is likely to retrieve sequence variants that are highly variable in length, such as the fungal ITS region, we developed an optimized workflow that is based on existing methods that are conveniently packaged.

**Figure 3:**
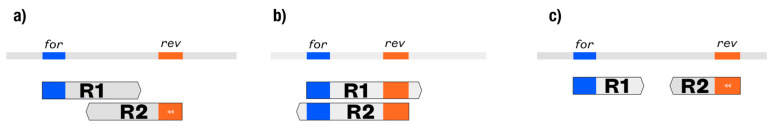
Effect of variable-length amplicons. (**a**) When the amplicon size is shorter than the sum of the two paired reads, it is possible to merge the fragments by their overlapping sequences. This is the most common setup in 16S amplicon sequencing. (**b**) When the target amplicon is shorter, each read will also sequence the opposite primer (primer read-through), requiring extra care in primer removal. (**c**) In case of longer amplicons, reads do not overlap, and thus cannot be merged. In this case, reads can be joined by introducing ‘Ns’ between the reads. Dadaist2 accommodates all three cases and is well-suited to variable amplicon lengths. *for*: forward primer; *rev*: reverse primer.

When the amplicon is short (Figure 3b), the sequences of one read can read across the primer of the opposite read (primer read-through); this situation is handled by the primer removal step. In the opposite scenario, when amplicons are longer than the sum of the two read lengths (Figure 3c), both reads will be processed separately, and only joined at the end of the workflow by connecting them with ‘Ns’. In a simulation, we show that, by joining the two reads, 53 unique ASVs could be recovered, which would otherwise have been missed. Taxonomic assignment was accurate when compared with the full sequence (Supplementary S4). This increases the information retrieved from long amplicons without decreasing the accuracy of taxonomic assignment. The typical 16S amplicon sequencing experiment does not require this specialized workflow, as the limited variability in length of the 16S rDNA variable regions allows to design experiments where the amplicon size is shorter than the sum of the length of the two paired-end reads (Figure 3a).

The opportunity to avoid joining the reads is provided by DADA2 via the option ‘justConcatenate’, but in this case even the sequences that are potentially merged remain concatenated. Moreover, this option is often neglected by wrappers (the popular DADA2 plugin for Qiime2, for example, does not allow enabling this feature). Dadaist2 makes use of this feature, and merges the overlapping pairs downstream while keeping the non-overlapping pairs concatenated. DADA2 users can process their output tables with the stand-alone ‘dadaist2-mergeseqs’ tool to achieve the same result.

#### 2.1.3. Modular Access to Individual Steps and User-Friendly Reports

Dadaist2 is designed to provide a set of modular command-line tools and wrappers and a whole pipeline. This modular design allows the reuse of some of the tools in custom pipelines or workflows, and the availability of the whole package in BioConda [22] makes it very easy to do so. For example, ‘dadaist2-assigntax’ can annotate a FASTA file generated by any tool. Similarly, ‘dadaist2-mergeseqs’ can process the feature table generated by DADA2 without joining the paired reads, generating both a new table and a reference FASTA file. To show this applicability on our website, we provide a NextFlow [23] example of a pipeline that uses various Dadaist2 modules.

Dadaist2 collects the execution logs of the tools used and combines them in a user-friendly HTML diagnostic report, which provides the user with an accessible overview of the analyses. The output of the primary analysis can also be rendered as a MultiQC [24] report summarizing the main findings. This includes the number of reads lost at each denoising step (filtering, denoising, merging, and chimera removal), the number of ASVs (and the sequence of the most abundant ones), and taxonomy bar plots, even before running any secondary analysis. We implemented the rendering of ‘octave plots’, histograms of abundances binned in logarithmic scale, as described by Edgar and Flyvbjerg [25]. This is extremely useful as it enables the user to inspect the experiment and evaluate whether any samples should be removed from the analysis, or to decide which downstream analyses to pursue.

### 2.2. Dadaist2 Secondary Analysis

One key feature of Dadaist2 is a framework for streamlining results from primary analysis results into the secondary analysis. Dadaist2 provides multiple interfaces for different user types, while focusing on reproducible and reliable methods in all pathways, implementing peer-reviewed and widely adopted procedures.

#### 2.2.1. Automatic Secondary Analyses from the Command Line Using Rhea

We adopted a publicly available peer-reviewed method called Rhea [12], which is based on R. Within Dadaist2, Rhea is supported both with automatic wrappers and by generating a directory of input files that are required by Rhea. Normalization, alpha diversity, and taxonomy binning steps are automatically performed, as they do not require any assumptions based on the metadata of the samples. Beta diversity and de novo clustering are also supported, provided that the users specify which metadata category to use for the analysis. This workflow can thus be performed automatically from the command-line, but also interactively in R following the Rhea protocol (to allow for custom adjustments).

#### 2.2.2. Interactive Secondary Analysis Using the MicrobiomeAnalyst Webserver

MicrobiomeAnalyst [13] is another tool to which we provide full support. It represents an easy-to-use web portal to interactively generate visualizations and statistical analyses of microbiome data. At the same time, MicrobiomeAnalyst maintains a high level of reproducibility, as the web service also provides the command history used to generate the results. Using MicrobiomeAnalyst does not require knowledge of R, though it can be run via RStudio, and thus is accessible to most scientists. The Dadaist2 workflow produces files that can be directly imported into MicrobiomeAnalyst allowing the user to easily dive into downstream data analyses and the creation of publication-ready figures.

#### 2.2.3. Custom Secondary Analyses Using PhyloSeq

For a flexible and interactive statistical analysis, the automatic Dadaist2 workflow saves the results from primary analysis, including the feature table, taxonomy, and phylogenetic tree, as a PhyloSeq object. This object is easily imported into R and can be analyzed using the PhyloSeq package, which offers several analytical methods and plotting functions. To facilitate code reuse and the propagation of reproducible methods, Dadaist2 allows macros based on a PhyloSeq object to be run directly from the command line. Some macros are distributed with the Dadaist2 repository to provide working examples, including the creation of abundance plots and alpha- and beta-diversity.

### 2.3. Examples and Validation

Dadaist2 relies on commonly adopted tools and provides a convenient set of tools to utilize them without introducing performance optimizations, and DADA2 denoising is at the core of the data processing provided by Dadaist2. Here we introduce the validation of our workflow to ensure its concordance with DADA2 natively run from R, and an example of a real-world dataset (from the ‘Mothur MiSeq SOP’) re-analyzed with Dadaist2 and confirming the findings of the original paper (where it was analyzed with a clustering approach using Mothur) [26,27].

#### 2.3.1. Validation and Comparison of Dadaist2 Components with Common Tools

To ensure the valid implementation of DADA2, we analysed the ‘Mothur MiSeq SOP’ (retrieved from https://mothur.org/wiki/miseq_sop/, accessed on 16 April 2021) and we analysed this using the same parameters used for DADA2 and Dadaist2. The results showed 100% overlap of identified ASVs and a near-identical community profile with 99.7% correlation between the two approaches (see S1 in the Supplementary file).

Furthermore, we tested Dadaist2 on a mock community sequenced in triplicate (from the ‘mockrobiota’ collection [28]), comparing it to Qiime2, using DADA2, and USEARCH (which implements an independent denoising algorithm). This revealed very similar profiles amongst all three tools, with the highest similarity between Qiime2 and Dadaist2, as expected (see S2 in the Supplementary file).

Validation of secondary analyses approaches was confirmed and showcased for the creation of a PhyloSeq object and composition plots from the mock community (see S3 in the Supplementary file).

#### 2.3.2. Reproduction of Biological Results on Real Data Using Dadaist2

**Figure 4:**
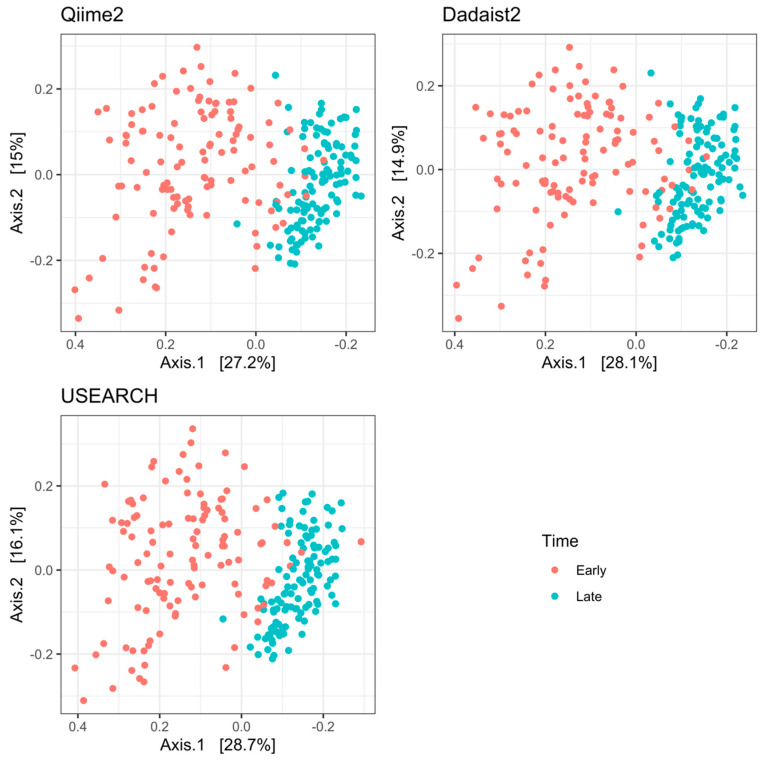
Principal Coordinate Analysis (PCoA) based on Bray-Curtis dissimilarity of faecal samples from a published dataset [26] analyzed using two popular tools providing an ASV-based result (USEARCH 11, using the Unoise3 algorithm, and Qiime2 2021.2, using the DADA2 algorithm that Dadaist2 also adopts) and with Dadaist2.

To showcase an example of Dadaist2’s applicability to real data, we reproduced the results of a previously published 16S amplicon study. We analysed the full dataset in Dadaist2, Qiime2 (version 2021.2) and USEARCH (version 11) and performed a PCoA analysis on the Bray–Curtis dissimilarities on taxonomic profiles of ‘early’ and ‘late’ time points (Figure 4), as described in the publication [26]. Even though amplicon approaches have advanced since the study, which was based on a clustering (OTU) approach, we reproduced the same biological result, showing a clear separation of the two sample types and a much larger variation between samples at the early timepoint. The correlation between the results from all three tools was significant (R function ‘procrustes’ from the vegan v2.5-6 package; significance: 0.001), but highest between Qiime2 and Dadaist2 (98%), whereas both tools were only 75% correlated with USEARCH. It should be noted that Dadaist2 required a single command to produce the representative sequences, the feature table, the taxonomic annotation and the PhyloSeq object that was used to produce the plots.

## 3. Discussion

Dadaist2 is a modular tool and pipeline for analysis of amplicon experiments, that bridges data analyses, visualization and numerical ecology. By building upon the excellent existing resources, Dadaist2 fills in the missing gap of easy transition between different analytical steps. The modular setup allows Dadaist2 to be used at different stages in the analyses. While it does provide an entire pipeline starting with raw sequencing data (to assist beginner users), experienced users can also import existing feature tables and taxonomic assignments from previous experiments or implement some of the provided tools in their existing workflow. This flexibility ensures the adaptability of workflows to a diversity of experiments, requirements, or collaborative efforts.

Recognizing that accurate numerical ecology is the foundation of comparable and reproducible amplicon studies, a key focus of Dadaist2 is to provide an easy link to statistical analyses in R. The implementation of existing tools (PhyloSeq, Rhea, and MicrobiomeAnalyst) was chosen to allow users from different backgrounds to perform statistical analyses and data visualization.

Dadaist2 further improves one major challenge in ITS sequencing. Variation in sequence length implies the need to account for amplicons that are either particularly long or short. Workflows had previously proposed methods to deal with short ITS sequences (see DADA2 tutorial: https://benjjneb.github.io/dada2/ITS_workflow.html, accessed on 10 March 2021, or as implemented in the very popular ‘q2-dada2’ plugin for Qiime2). In contrast, Dadaist2 also implements a newly designed workflow that handles long amplicon sequences. This is commonly not specifically accounted for, but is essential as microbiome studies increasingly include the important, yet often neglected, non-bacterial fraction of microbial communities [29].

The development of a pipeline requires several factors and trade-offs to be balanced. For example, ease of use alongside the opportunity to fine-tune each setting, and to achieve ease of installation and executability in various environments. Dadaist2 excels in the ease of installation and execution, with the one-step installation via Miniconda and the availability of Docker and Singularity containers. This is complemented by detailed, openly accessible documentation and follow-along tutorials. Additionally, we implemented the easy use of integrated wrappers, balanced by the flexibility of the atomic commands used in pre-existing pipelines. For example, our new implementation of modules, such as the crosstalk removal algorithm or handling of variable amplicon lengths, can easily be used in any metabarcoding experiment. One key element of Dadaist2 is to facilitate easily accessible secondary analyses. Overcoming the tedious step of data-reformatting and change in coding language, this process is automatically performed by the pipeline. This allows both interactive and easy data exploration via MicrobiomeAnalyst, and offline non-interactive analyses via implementation of the Rhea pipeline or using custom scripts based on Rhea or PhyloSeq. Dadaist2 allows users with different levels of coding experience to achieve state-of-the-art amplicon experiments and keep in touch with their data throughout all analyses.

## 4. Materials and Methods

The pipeline is freely available from GitHub (https://github.com/quadram-institute-bioscience/dadaist2, accessed on 17 April 2021) and its online documentation is available from the website (https://quadram-institute-bioscience.github.io/dadaist2/, accessed on 17 April 2021).

The Dadaist2 main pipeline and the command line wrappers are written in Perl (requires Perl 5.12), and a collection of R scripts are used in the background. The crosstalk removal tool has been implemented in Python (using Pandas). The pipeline requirements (DADA2, Decipher, PhyloSeq, Biom-Format, Clustal Omega [30], Cutadapt, Fastp, FastTree [31], Pandas, SeqFu [32], Qax [33]) are installed automatically using Miniconda, as described in the documentation.

Dadaist2 is available for installation via Miniconda, Docker or Singularity container, and thus is easy to execute in High-Performance Computing clusters.

To test the pipeline, we used a 16S and an ITS dataset [34,35], a small subset of which is provided with the software in the repository.

## Figures and Tables

**Table 1:** Comparison of the features of command-line tools wrapping DADA2 to denoise amplicon reads: Dadaist2, DadaSnake and Qiime2 [1,2].

Feature	Dadaist2	DadaSnake	Qiime2
Command Line Interface	✔	✔	✔
Modular design	✔	—	✔
Framework with plugins	—	—	✔
Alternative denoisers to DADA2	—	—	✔
Easy installation	✔	—	✔
Start to End pipeline	✔	✔	—
Crosstalk reduction	✔	—	—
MultiQC summary report	✔	—	—
Export to Rhea	✔	—	—
Export to Microbiome Analyst	✔	—	—
Export to PhyloSeq	✔	✔	—

## Data Availability

Source code is freely available from GitHub, at https://www.github.com/quadram-institute-bioscience/dadaist2, accessed on 17 April 2021. An archive of the published version has been deposited to Zenodo (http://doi.org/10.5281/zenodo.4761408, accessed on 14 May 2021).

## Supplementary Materials

The following are available online at https://www.mdpi.com/article/10.3390/ijms22105309/s1, Supplementary paragraph S1: Technical validation of DADA2 embedding; Supplementary paragraph S2: Analysis of Fungal Mock Community (ITS); Supplementary paragraph S3: Reproducibility of R commands automation; Supplementary paragraph S4: Long amplicons;
