# Supplementary material for: Dadaist2: A Toolkit to Automate and Simplify Statistical Analysis and Plotting of Metabarcoding Experiments
Source: Int J Mol Sci. 2021 May 18;22(10):5309. doi: 10.3390/ijms22105309 (PMC8157834; doi:10.3390/ijms22105309)
Supplement: Supplementary file 1 [file ijms-22-05309-s001.zip › ijms-1208613-supplementary.pdf]

S1. Technical validation of DADA2 embedding

Dadaist2 is a workflow that relies on the DADA2 R package. We implemented a suite of consistency checks to test that the system calls to external tools produce reliable results. The suite is run at each release to ensure that the pipeline continues providing reliable results.

DADA2 denoising process is modeled after the DADA2 workflow as presented in the package documentation available at: https://benjjneb.github.io/dada2/tutorial_1_8.html (visited 16 April 2021). We ran DADA2 from R using a small dataset, and then compared the results produced by Dadaist2. Dadaist2 can automatically select the trimming, or let the user manually input the trimming boundaries. To ensure the comparison is not affected by difference in trimming length, we use the same parameter both for DADA2 and Dadaist2.

S1.1 The input dataset

To run DADA2 programmatically we wrote the workflow from the tutorial in an R script (available from the respository, called `dada2-sop.R`) and invoke it as:

```
mkdir dada2-vanilla
Rscript ./test/miseq-sop-compare/dada2-sop.R \
  subsample/ dada2-vanilla 231 215
```

Dadaist2 (0.8.0 and subsequent releases) was tested with this command (where we skip the initial QC to feed the truncation boundaries):

```
dadaist2 -i subsample/ \
 -o dadaist_output \
 -r ~/volume/dadaist2/refs/silva_nr_v138_train_set.fa.gz \
 -t 30 --skip-qc --trunc-len-1 231 --trunc-len-2 215
```

The test ensures that the two programs identify the same ASVs by clustering the two outputs using cd-hit-est and checking that all the clusters contain one sequence from Dadaist2 and one sequence from DADA2. The pipeline is in the repository (in `/validation/mothur-sop/do-compare.sh`).

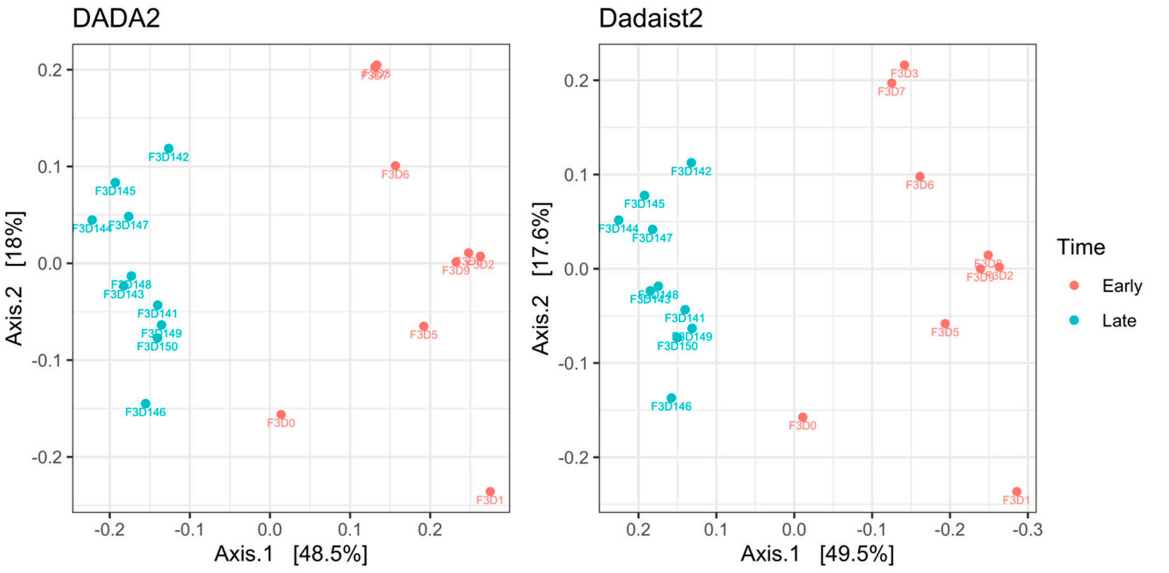

Supplementary Figure S1: PCoA of DADA2 and Dadaist2 results on the "MiSeq SOP" dataset

S2. Analysis of Fungal Mock Community (ITS)

S2.1 The dataset

The dataset comes from the Mockrobiota collection (https://github.com/caporaso-lab/mockrobiota), namely sample **mock-9**, composed by 16 fungal strains (**12 species**) and originally published in Bokulich 2015, and composed by three samples with the same composition (barcodes AAGTGGCTATCC, GTTCACGCCCAA and CGTTCCTTGTTA).

The reads and the associated metadata are available at:

- https://github.com/caporaso-lab/mockrobiota/tree/master/data/mock-9/sample-metadata.tsv
- https://s3-us-west-2.amazonaws.com/mockrobiota/latest/mock-9/mock-forward-read.fastq.gz
- https://s3-us-west-2.amazonaws.com/mockrobiota/latest/mock-9/mock-reverse-read.fastq.gz
- https://s3-us-west-2.amazonaws.com/mockrobiota/latest/mock-9/mock-index-read.fastq.gz

The quality scores have been converted to the Illumina 1.8 encoding:

```
INPUT="@ABCDEFGHIJKLMNOPQRSTUVWXYZ[\\]^_`abcdefghi"
sed -i -e '4~4y/$INPUT/="!"#$%&'\''()*+,-.\/0123456789:;<=>?@ABCDEFGHIJ/' *.fastq
```

The counts on the demultiplexed samples are:

```
TAACAAGGAACG_S0_L001_R1_001.fastq.gz    16348   Paired
GGATAGCCACTT_S0_L001_R1_001.fastq.gz    31629   Paired
TTGGGCGTGAAC_S0_L001_R1_001.fastq.gz    31681   Paired
```

S2.2 Processing with Qiime2, USEARCH and Dadaist2

The reads from the three samples have been analysed using Qiime2 (version 2020.2), USEARCH (version 11) and Dadaist2 (version 0.8).

Qiime 2 (2020.2) workflow was performed as follows:

```
qiime tools import \
  --type 'SampleData[SequencesWithQuality]' \
  --input-path reads \
  --input-format CasavaOneEightSingleLanePerSampleDirFmt \
  --output-path reads.qza

qiime dada2 denoise-paired  \
  --i-demultiplexed-seqs reads.qza  \
  --o-table table-dada2.qza  \
  --o-representative-sequences rep-seqs-dada2.qza \
  --p-trim-left-f 0  --p-trim-left-r 0  \
  --p-trunc-len-f 0  --p-trunc-len-r 0  \
  --p-n-threads 32   --p-n-reads-learn 10000 \
  --o-denoising-stats dada2-stats.qza

qax x -o ./ dada2-stats.qza table-dada2.qza
biom convert --to-tsv -i table-dada2.biom -o table.tsv
sed -i '/Constructed from biom/d' table.tsv
```

Dadaist2 was run with default parameters:

```
dadaist2 -i reads/ -o dadaist-output/
```

The “unoise” (version 3) algorithm from USEARCH is used in a workflow as follows:

```
usearch -fastq_mergepairs *R1*.fastq -relabel @ -fastqout merge.fastq
usearch -fastq_filter merge.fastq -fastq_maxee 0.4 -fastaout filt.fa
usearch -fastx_uniques filt.fa -fastaout uniq.fa -sizeout -relabel uniq.
usearch -unoise3 uniq.fa -zotus asv.fa
usearch -otutab merge.fastq -db asv.fa -otutabout table.tsv
```

S2.3 Results

The three pipelines identified a similar number of ASVs:

- Unoise (v3) 11
- Qiime2 2021.2 13
- Dadaist2 v0.8 12

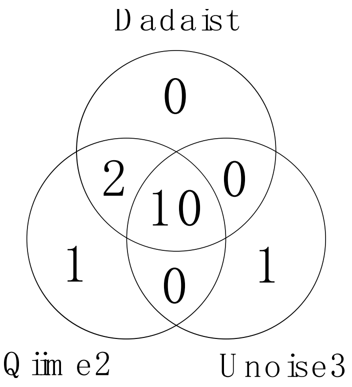

Clustering the sequences (with cd-hit-est) at 100% identity, 10 are shared among all the pipelines, and 2 are shared between Qiime2 and Dadaist2 (as reasonably expected since they both rely on DADA2). Dadaist2, with default parameters, identified 12 ASVs in the mock community correctly representing the composition.

The differences are not only negligible in number, but also refers to sequences that are very rare in the dataset (hence minimal difference in the parameters will lead to their inclusion/exclusion from the final denoised dataset). The following bar plot shows the abundance in each sample of the representative sequences, where the shared ones are coloured and the unshared are in grey. It is expected to note a higher concordance among Qiime2 and Dadaist2, as they both used DADA2 to perform the denoising, where Unoise has been added to show the good overlap with a different approach.

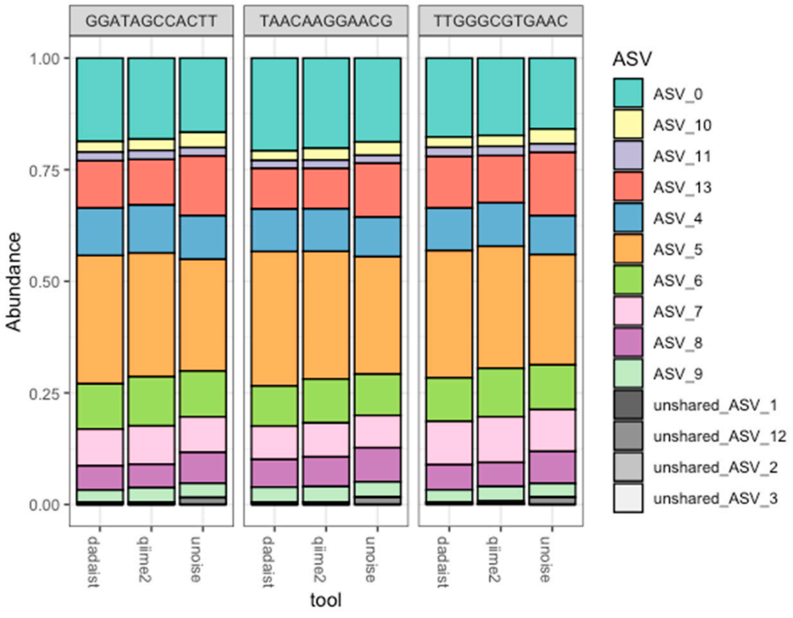

Supplementary Figure S2: Composition of three mock community samples analyzed with Qiime2, Dadaist2 and Unoise (from USEARCH).

Taxonomy classification of the ASV identified by Dadaist2, performed with **dadaist2-assigntax** using UNITE[1]:

	Phylum	Class	Order	Family	Genus	Species
1	Ascomycota	Saccharomycetes	Saccharomycetales	Saccharomycetales_ic	NA	NA
2	Ascomycota	Saccharomycetes	Saccharomycetales	Saccharomycetaceae	Issatchenkia	Issatchenkia_orientalis
3	Ascomycota	Saccharomycetes	Saccharomycetales	Dipodascaceae	unidentified	Dipodascaceae_sp
4	Ascomycota	Saccharomycetes	Saccharomycetales	Debaryomycetaceae	Hyphopichia	Hyphopichia_burtonii
5	Ascomycota	Saccharomycetes	Saccharomycetales	Dipodascaceae	Dipodascus	Dipodascus_geotrichum
6	Ascomycota	Saccharomycetes	Saccharomycetales	Dipodascaceae	NA	NA
7	Ascomycota	Saccharomycetes	Saccharomycetales	Dipodascaceae	unidentified	Dipodascaceae_sp
8	Ascomycota	Saccharomycetes	Saccharomycetales	Dipodascaceae	Dipodascus	Dipodascus_geotrichum
9	Ascomycota	Saccharomycetes	Saccharomycetales	Dipodascaceae	Dipodascus	Dipodascus_geotrichum
10	Ascomycota	Saccharomycetes	Saccharomycetales	Dipodascaceae	NA	NA
11	Ascomycota	Saccharomycetes	Saccharomycetales	Saccharomycetaceae	Issatchenkia	Issatchenkia_orientalis
12	Ascomycota	Saccharomycetes	Saccharomycetales	Saccharomycetaceae	Issatchenkia	Issatchenkia_orientalis

[1] https://github.com/quadram-institute-bioscience/dadaist2/releases/download/v0.7.3/uniref.fa.gz

S3. Reproducibility of R commands automation

Dadaist2 relies on several R libraries both for the primary (DADA2, DECIPHER) and for the secondary (PhyloSeq, vegan, *etc*.) analysis.
We designed Dadaist2 around the concept of separating user logic from R commands, so that the wrappers are responsible to collect input parameters, checking their consistency and the existence of required file, and then to execute an external R script. In this way the possibility to redo the steps in R is guaranteed.
The primary analysis is a mix of R and non-R components, and thus required an extensive validation that we showed on Supplementary data S1. In this paragraph we selected a typical secondary workflow and compared the result of the automation and the manual execution of the commands.

Workflow

Most seconday analyses are based on a PhyloSeq object that Dadaist2 creates automatically, so our workflow starts by importing the required files (feature table, representative sequences, metadata…) into a PhyloSeq object, and then we use the created object to plot the taxonomy as stacked barchart and as bubble plot (the former being a native PhyloSeq plot, the latter a custom implementation we added in Dadaist2). Within Dadaist2, this functionality can be accessed via the dadaist2-taxplot wrapper (that calls the accessory *D2-AbundancesPhyloseq.R* script) that uses as input the PhyloSeq object generated by the primary analysis:

```
dadaist2-taxplot -i phyloseq.rds -o ./plots/
```

To test if manual creation of a phyloseq object and usage of phyloseq will produce identical results, we created a R markdown performing each step manually. This markdown containing the manual R worfklow can be is available from the GitHub repository at the address: https://quadram-institute-bioscience.github.io/dadaist2/notes/plot.html (accessed 10 May 2021).
The procedure and the results are also available in the documentation at the address https://quadram-institute-bioscience.github.io/dadaist2/notes/6_Rscripts.html (accessed 10 May 2021).

Results

The phyloseq objects created automatically by Dadaist2 and manually from the raw data output are identical in the numbers of taxa, samples, variables, tree features and taxonomic ranks (Supplementary Figure S3). Similarly, the bar charts and bubble plots created by Dadaist2-implemented script D2-AbundancesPhyloseq.R from the automatically created phyloseq object and the manual creation of the same plots produce idential results (See examples in Supplementary Figurres S4, S5).

```
# Print phyloseq object
my_physeq
```

```
## phyloseq-class experiment-level object
## otu_table()   OTU Table:         [ 218 taxa and 20 samples ]
## sample_data() Sample Data:       [ 20 samples by 2 sample variables ]
## tax_table()   Taxonomy Table:    [ 218 taxa by 8 taxonomic ranks ]
## phy_tree()    Phylogenetic Tree: [ 218 tips and 216 internal nodes ]
```

```
# Compare phyloseq object to automatically created one from dadaist
my_physeq_auto<-readRDS('../dadaist_0.9.0/R/phyloseq.rds')
print(my_physeq_auto)
```

```
## phyloseq-class experiment-level object
## otu_table()   OTU Table:         [ 218 taxa and 20 samples ]
## sample_data() Sample Data:       [ 20 samples by 2 sample variables ]
## tax_table()   Taxonomy Table:    [ 218 taxa by 8 taxonomic ranks ]
## phy_tree()    Phylogenetic Tree: [ 218 tips and 216 internal nodes ]
```

Supplementary Figure S3: Output from comparison of manually created phyloseq object (top) and automatically created and imported phyloseq object from Dadaist2 (bottom) highlighting identical numbers of taxa, samples and taxonomic ranks.

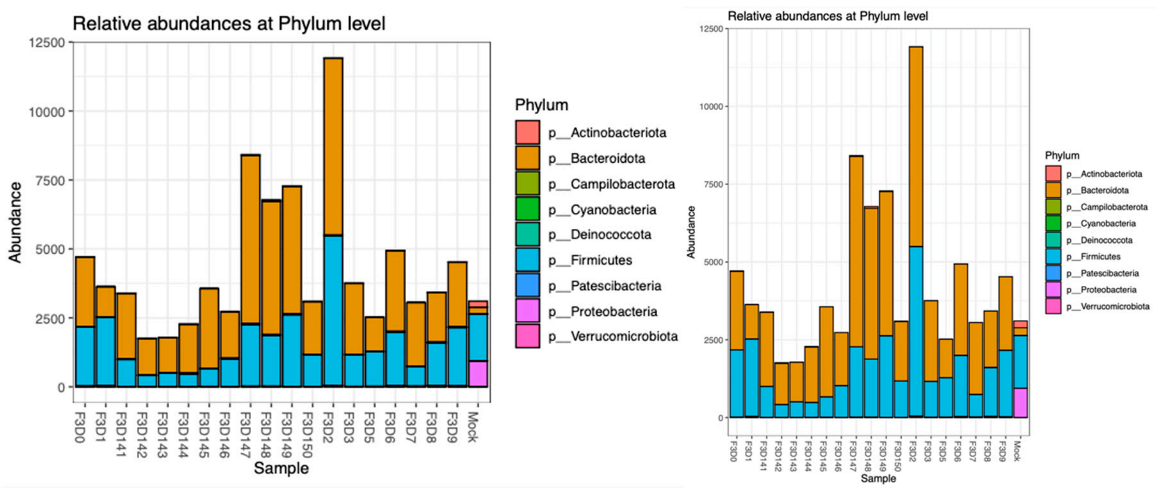

Supplementary Figure S4: Bar charts created manually with phyloseq and its *plot_bar* function (left); and created automatically from Dadaist2 phyloseq object using *dadaist2-taxplot* wrapper (that calls the accessory *D2-AbundancesPhyloseq.R* script) (right).

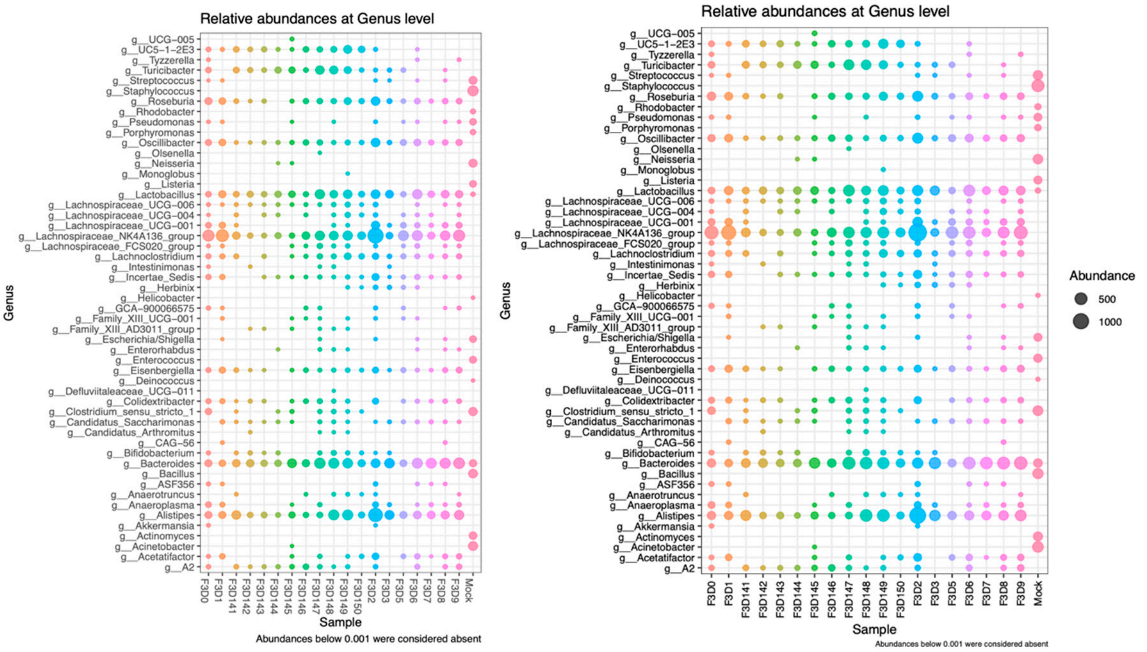

Supplementary Figure S5: Bubble plots created manually with phyloseq and a custom ggplot2 implementation (left) and created automatically from the Dadaist2 phyloseq object using dadaist2-taxplot wrapper (that calls the accessory *D2-AbundancesPhyloseq.R* script) (right).

S4. Long amplicons

Taxonomic annotation of joined representative sequences

Dadaist2 implements a dedicated approach to handle amplicon lengths that exceed the combined length of both read pairs. If this is the case, reads cannot be merged to cover the entire target region. Despite this, the paired-read information can still be used to retrieve most of the amplicon. To do so, the module processes paired reads independently until the end of the workflow, when they are joined by 'Ns' into a single sequence. This sequence is used for taxonomic classification.
To test whether taxonomic classification is sensitive and accurate we simulated a 600bp long target region sequenced with 2x300bp MiSeq reads. In this case some reads could not be merged as they did not overlap by 20bp or more. In total we identified 53 unique sequences that were longer than 550bp. These sequences would have been missed if relying only on merged reads. By joining these reads, and filling in the gap with 'Ns', we could thus recover 53 ASV that would otherwise have been missed.
To test the accuracy of taxonomic assignment based on the joined-read approach, we compared their taxonomic assignment to sequences that covered the entire amplicon (Figure A1). The test showed that joined reads were as accurate in their taxonomic assignment as the full amplicon sequence. Taxonomic assignment further revealed that 38 genus-level taxa could be recovered.
By using this approach paired reads can still be used, even when amplicon sequences are too long to be fully covered by the reads; this increases the overall sensitivity in profiling of the community composition.

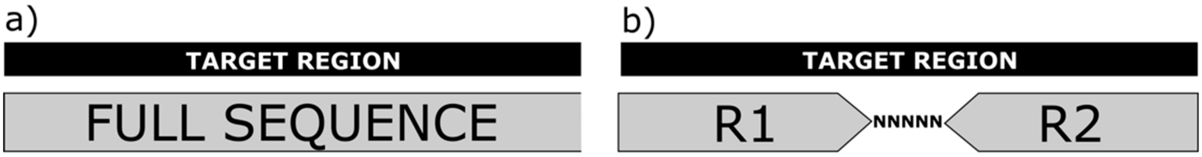

Figure S4 – Simulation test scenario of full sequence coverage of the target region (a) compared with joined read pairs (b). Scenario a is not achievable when the amplicon is too long, but the approach in b is sufficient for accurate taxonomic assignment.

The results and data of the simulation are documented in the software repository https://quadram-institute-bioscience.github.io/Dadaist/notes/2_ITS.html (6 May 2021).

Impact on the number of Ns used

To assess the impact of the number of Ns used to separate the two paired ends, we made a dedicated simulation to compare the taxonomy assigned to non-overlapping reads.
DADA2 arbitrarily insert 6 Ns between the joined sequences. We joined the simulated pairs with 0, 1, 6, 12 and 100 Ns, respectively, then we assigned the taxonomy with the same method (using *dadaist2-assigntax* against the same UNITE database used to generate the dataset).
The full procedure used to perform this evaluation is available in the GitHub repository (), in particular a script called *compare-tax.pl* has been implemented to compare the assigned taxonomies as:

```
paste {0,1,6,12,100}Ns/taxonomy.tsv | ./compare-tax.pl
```

The full output of the script is reported below, showing that all the assigned taxonomy have been 100% identical.

```
<OK> 1 Fungi Ascomycota Leotiomycetes Helotiales Myxotrichaceae Oidiodendron Oidiodendron_sp
<OK> 2 Fungi Ascomycota Sordariomycetes Chaetosphaeriales Chaetosphaeriaceae Chloridium Chloridium_paucisporum
<OK> 3 Fungi Ascomycota Leotiomycetes Helotiales Dermateaceae Pseudofabraea Pseudofabraea_citricarpa
```

<OK> 4 Fungi Ascomycota Leotiomycetes Helotiales unidentified unidentified Helotiales_sp

<OK> 5 Fungi Ascomycota Eurotiomycetes Phaeomoniellales Phaeomoniellaceae Pseudophaeomoniella Pseudophaeomoniella_oleae

<OK> 6 Fungi Ascomycota Leotiomycetes Helotiales Dermateaceae Parafabraea Parafabraea_caliginosa

<OK> 7 Fungi Basidiomycota Cystobasidiomycetes Erythrobasidiales Erythrobasidiaceae Bannoa Bannoa_ogasawarensis

<OK> 8 Fungi Ascomycota Lecanoromycetes Lecanorales Cladoniaceae Cladonia Cladonia_polyscypha

<OK> 9 Fungi Ascomycota Orbiliomycetes Orbiliales Orbiliaceae Orbilia Orbilia_rectispora

<OK> 10 Fungi Ascomycota Dothideomycetes Pleosporales Thyridariaceae Roussoella Roussoella_elaeicola

<OK> 11 Fungi Ascomycota Leotiomycetes Helotiales Hyaloscyphaceae Lachnum Lachnum_asiaticum

<OK> 12 Fungi Ascomycota Leotiomycetes Helotiales Hyaloscyphaceae Lachnum Lachnum_asiaticum

<OK> 13 Fungi Ascomycota Leotiomycetes Helotiales Dermateaceae Calloria Calloria_urticae

<OK> 14 Fungi Ascomycota Saccharomycetes Saccharomycetales unidentified unidentified Saccharomycetales_sp

<OK> 15 Fungi Ascomycota Leotiomycetes Helotiales Chrysodiscaceae Chrysodisca Chrysodisca_peziculoides

<OK> 16 Fungi Ascomycota Orbiliomycetes Orbiliales Orbiliaceae unidentified Orbiliaceae_sp

<OK> 17 Fungi Ascomycota Eurotiomycetes Chaetothyriales unidentified unidentified Chaetothyriales_sp

<OK> 18 Fungi Ascomycota Eurotiomycetes Chaetothyriales unidentified unidentified Chaetothyriales_sp

<OK> 19 Fungi Ascomycota Orbiliomycetes Orbiliales Orbiliaceae Dactylella Dactylella_cylindrospora

<OK> 20 Fungi Ascomycota Eurotiomycetes Eurotiales Trichocomaceae Rasamsonia Rasamsonia_sp

<OK> 21 Fungi Ascomycota Leotiomycetes Helotiales Helotiales_fam_Incertae_sedis Chalara Chalara_longipes

<OK> 22 Fungi Ascomycota Pezizomycetes Pezizales Sarcosomataceae Donadinia Donadinia_seaveri

<OK> 23 Fungi Ascomycota Orbiliomycetes Orbiliales Orbiliaceae Orbilia Orbilia_carpoboloides

<OK> 24 Fungi Ascomycota Orbiliomycetes Orbiliales Orbiliaceae Orbilia Orbilia_sp

<OK> 25 Fungi Ascomycota Eurotiomycetes Chaetothyriales unidentified unidentified Chaetothyriales_sp

<OK> 26 Fungi Ascomycota Leotiomycetes Helotiales Hyaloscyphaceae Incrucipulum Incrucipulum_sulphurellum

<OK> 27 Fungi Ascomycota Orbiliomycetes Orbiliales Orbiliaceae Orbilia Orbilia_aprilis

<OK> 28 Fungi Ascomycota Eurotiomycetes Chaetothyriales Herpotrichiellaceae Cladophialophora Cladophialophora_sp

<OK> 29 Fungi Ascomycota Orbiliomycetes Orbiliales Orbiliaceae Hyalorbilia Hyalorbilia_sp

<OK> 30 Fungi Ascomycota Eurotiomycetes Chaetothyriales unidentified unidentified Chaetothyriales_sp

<OK> 31 Fungi Ascomycota Orbiliomycetes Orbiliales Orbiliaceae unidentified Orbiliaceae_sp

<OK> 32 Fungi Ascomycota Leotiomycetes Helotiales Helotiaceae Hymenoscyphus Hymenoscyphus_sp

<OK> 33 Fungi Ascomycota Leotiomycetes Helotiales Leotiaceae Pezoloma Pezoloma_ericae

<OK> 34 Fungi Ascomycota Orbiliomycetes Orbiliales Orbiliaceae Orbilia Orbilia_sp

<OK> 35 Fungi Ascomycota Eurotiomycetes Chaetothyriales unidentified unidentified Chaetothyriales_sp

<OK> 36 Fungi Ascomycota Dothideomycetes Capnodiales Mycosphaerellaceae Scleroramularia Scleroramularia_pomigena

<OK> 37 Fungi Ascomycota Sordariomycetes Xylariales Xylariaceae Annulohypoxylon Annulohypoxylon_stygium

<OK> 38 Fungi Ascomycota Geoglossomycetes Geoglossales Geoglossaceae Geoglossum Geoglossum_sp

<OK> 39 Fungi Ascomycota Leotiomycetes Helotiales Helotiales_fam_Incertae_sedis Vestigium Vestigium_sp

<OK> 40 Fungi Ascomycota Leotiomycetes Helotiales Helotiaceae Phaeohelotium Phaeohelotium_sp

<OK> 41 Fungi Ascomycota Leotiomycetes Helotiales Hyaloscyphaceae Capitotricha Capitotricha_bicolor

<OK> 42 Fungi Ascomycota Orbiliomycetes Orbiliales Orbiliaceae Dactylella Dactylella_leptospora

<OK> 43 Fungi Basidiomycota Agaricomycetes Russulales Lachnocladiaceae Dichostereum Dichostereum_durum

<OK> 44 Fungi Ascomycota Eurotiomycetes Chaetothyriales unidentified unidentified Chaetothyriales_sp

<OK> 45 Fungi Basidiomycota Agaricomycetes Hymenochaetales Hymenochaetaceae Coltricia Coltricia_sp

<OK> 46 Fungi Ascomycota Eurotiomycetes Chaetothyriales unidentified unidentified Chaetothyriales_sp

<OK> 47 Fungi Ascomycota Eurotiomycetes Chaetothyriales unidentified unidentified Chaetothyriales_sp

<OK> 48 Fungi Ascomycota Eurotiomycetes Chaetothyriales Herpotrichiellaceae Exophiala Exophiala_sp

<OK> 49 Fungi Ascomycota Dothideomycetes Asterinales Asterinaceae Blastacervulus Blastacervulus_eucalypti

<OK> 50 Fungi Basidiomycota Agaricomycetes Polyporales unidentified unidentified Polyporales_sp

<OK> 51 Fungi Mucoromycota Endogonomycetes GS22 unidentified unidentified GS22_sp

<OK> 52 Fungi Ascomycota Orbiliomycetes Orbiliales Orbiliaceae Orbilia Orbilia_sp

<OK> 53 Fungi Ascomycota Sordariomycetes Chaetosphaeriales Chaetosphaeriaceae Chloridium Chloridium_paucisporum
